# A Bibliometric Analysis of the Top 100 Most‐Cited Articles on Skin Photoaging

**DOI:** 10.1111/jocd.70119

**Published:** 2025-03-12

**Authors:** Yan Teng, Hui Tang, Yibin Fan, Jianhua You

**Affiliations:** ^1^ Center for Plastic & Reconstructive Surgery, Department of Dermatology Zhejiang Provincial People's Hospital, Affiliated People's Hospital, Hangzhou Medical College Hangzhou Zhejiang China; ^2^ Dermatology Hospital of Zhejiang Province Huzhou Zhejiang China

**Keywords:** bibliometric analysis, citation, Most‐cited articles, skin photoaging, VOS viewer, web of science

## Abstract

**Background:**

In recent decades, our understanding of the pathogenesis and pathophysiology of skin photoaging has improved considerably, thereby enhancing preventive and management strategies. The bibliometric analysis demonstrates the chronological trends of publications, highlighting the most influential studies related to skin photoaging.

**Objective:**

This study aims to identify and analyze the top 100 most‐cited articles related to skin photoaging to offer bibliometric information.

**Methods:**

The Web of Science database was searched to obtain publications on skin photoaging. Information from the top 100 most‐cited articles was extracted and analyzed using Microsoft Excel 2019 and VOSviewer (version 1.6.18).

**Results:**

The top 100 most‐cited articles on skin photoaging received a total of 23 925 citations and an average of 239 citations per article. The top most‐cited article received 1019 citations and 30.88 citations per article. The publication year ranged from 1995 to 2010, with a peak period of top publications between 2001 and 2005. *The Journal of Investigative Dermatology* and the *Journal of the American Academy of Dermatology* published the largest number of top‐cited articles. The articles originated from nine different countries, with The United States as the highest contributor. Fisher GJ was the most productive first author from the University of Michigan in the United States and published a total of three articles. A total of 62 keywords were included and grouped into three clusters: ‘matrix metalloproteinase’, ‘collagen’ and ‘radiation’. The article types include only reviews and original articles. Prevention and treatment‐related studies were the most common research focus, followed by pathogenesis, pathophysiology, clinical features and screening methods.

**Conclusion:**

This bibliometric analysis on skin photoaging demonstrated a major upward trend in the prevention and treatment of skin photoaging, which provides a foundation for future research.

## 1 Introduction

Photoaging is defined as the premature aging of the skin due to prolonged solar exposure, which mainly consists of ultraviolet radiations (UVR), visible light, and infrared rays [1]. Recently, visible light, especially blue light, has been reported to induce skin photoaging; however, UVR is also considered a major etiology [2, 3]. The common clinical manifestations of skin photoaging include wrinkles, discoloration, telangiectasias, skin dehydration, and rough appearance, which are associated with the pathophysiological changes of different tissue cells in both the epidermis and dermis. For example, wrinkles, a common clinical feature of photoaging, are mainly induced by a decrease in dermal fibroblasts and a faster breakdown rate compared to the slower collagen and elastin synthesis rates [4].

Multiple pathways are involved in the pathogenesis of photoaging induced by UVR, which directly damage DNA, RNA, and proteins and indirectly damage these components via immunosuppression and inflammation, accumulation of matrix metalloproteinases (MMPs) and reduction of collagens [5]. Over the last few decades, along with the public's pursuit of aesthetic appearances, research on skin photoaging has been prevailing.

Citation analysis is the most commonly used bibliometric method to quantify published articles. A detailed and thorough bibliometric analysis of the most‐cited publications could be utilized to highlight the development of a specific discipline and the future research direction of a specific field. Influential articles related to skin photoaging are an important source of evidence‐based outcomes that could be identified and analyzed via citation analysis [6]. Multiple bibliometric analyses have been utilized in the field of dermatology [7]. Sun et al. [8] conducted a bibliometric analysis to investigate the landscape of photoaging during the recent two decades. However, there is a lack of bibliometric analysis of the top most‐cited publications on skin photoaging to the best of our knowledge. Therefore, this study aims to identify the top 100 most‐cited articles in skin photoaging, analyze the bibliometric information, and lay the foundation for future research.

## 2 Methods

### 2.1 Searching Strategy

On May 2024, the Web of Science (WOS) database was utilized for citations of all the published articles on skin photoaging. No time limitation was set for the research and both the original articles and review articles were included. Our main keywords for the research “skin photoaging” or “photoaging” or “photoaging of skin” or “solar aging of skin” were found using MESH database. Keywords were searched in the title, abstract and keywords of the articles. Artiles were firstly assessed for their suitability for inclusion by title and in case of ambiguity, the abstracts and full texts were also reviewed. The searched articles were ranked according to their citation count in descending order. Articles that focus majorly on ‘skin photoaging’ were included whereas those that focused solely on other topics were excluded.

### 2.2 Data Extraction and Statistical Analysis

VOS viewer was used for visualizing the network of occurrence of keywords in the top 100 most‐cited articles. Additionally, we used Microsoft Excel 2019 to analyze the data from the WOS database and to construct the figures and tables. Two researchers (Yan Teng and Hui Tang) independently and carefully screened all the articles. They discussed the differences until a consensus was reached. All data were downloaded from the WOS database, and VOS viewer (version 1.6.18) and Microsoft Excel 2021were used to extract and analyze article information, including title, author listing, journal name (impact factors), publishing year, citation count (CC), citation per year (CY) and country of origin. Journal impact factors were included in the 2022 Incites Journal Citation Reports. IBM SPSS Statistics 26.0 was used to apply tests of significance. Correlation between the impact factor and the total number of citations of a journal was also assessed. For all the statistical tests, a p‐value of < 0.05 was considered significant. Ethical approval was not needed for the study since it was limited to analyzing previously published data.

## 3 Results

A total of 2868 articles were selected based on the CC. The top 100 most‐cited articles are listed in Table 1.

**TABLE 1:** The top 100 articles on skin photoaging.

Rank	Title	First Author	Journal	Year of publication	CC	CY
1	Mechanisms of photoaging and chronological skin aging	Fisher GJ	Archives of dermatology	2002	1136	51.63
2	Fractional photothermolysis: A new concept for cutaneous remodeling using microscopic patterns of thermal injury	Manstein D	Lasers in surgery and medicine	2004	1060	53
3	Pathophysiology of premature skin aging induced by ultraviolet light	Fisher, GJ	The New England journal of medicine	1997	1023	37.89
4	Toxic effects of ultraviolet radiation on the skin	Matsumura Y	Toxicology and applied pharmacology	2004	857	42.85
5	UV‐induced skin damage	Ichihashi, M	Toxicology	2003	725	34.52
6	Wound heating effect of adipose‐derived stem cells: A critical role of secretory factors on human dermal fibroblasts	Won‐Serk Kim	Journal of dermatological science	2007	659	38.76
7	Role of Matrix Metalloproteinases in Photoaging and Photocarcinogenesis	Pittayapruek P	International journal of molecular sciences	2016	631	78.875
8	Oxidation events and skin aging	Kammeyer A	Aging research reviews	2015	540	60
9	Pulsed carbon dioxide laser resurfacing of photoaged facial skin	Fitzpatrick RE	Archives of dermatology	1996	436	15.57
10	Vitamin A antagonizes decreased cell growth and elevated collagen‐degrading matrix metalloproteinases and stimulates collagen accumulation in naturally aged human skin	J Varani	The Journal of investigative dermatology	2000	409	12.39
11	Oxidation events and skin aging	Kammeyer A	Aging research reviews	2015	385	11.67
12	Antioxidant activity, lipid peroxidation and skin diseases. What's new	S Briganti	Journal of the European Academy of Dermatology and Venereology	2003	382	11.58
13	Photoaging: Mechanisms and repair	Rabe JH	Journal of the American Academy of Dermatology	2006	339	10.27
14	UV‐induced reactive oxygen species in photocarcinogenesis and photoaging	Scharffetter‐Kochanek K	Biological chemistry	1997	337	10.21
15	Cutaneous photodamage, oxidative stress, and topical antioxidant protection	Pinnell SR	Journal of the American Academy of Dermatology	2003	332	10.06
16	Skin aging and photoaging—an overview	Gilchrest BA	Journal of the American Academy of Dermatology	1989	323	9.79
17	Mitochondrial redox control of matrix metalloproteinases	Nelson KK	Free radical biology & medicine	2004	319	9.67
18	Photoaging of the skin from phenotype to mechanisms	Scharffetter‐Kochanek K	Experimental gerontology	2000	315	9.55
19	Ultraviolet‐B irradiation and matrix metalloproteinases—From induction via signaling to initial events	Brenneisen P	Annals of the New York Academy of Sciences	2002	314	9.52
20	Solar UV irradiation and dermal photoaging	Wlaschek M	Journal of photochemistry and photobiology. B, Biology	2001	310	9.39
21	Intrinsic aging vs. photoaging: a comparative histopathological, immunohistochemical, and ultrastructural study of skin	M El‐Domyati	Experimental dermatology	2002	309	9.36
22	Advanced glycation end products Key players in skin aging?	Paraskevi Gkogkolou	Dermato‐endocrinology	2012	304	9.21
23	Retinoic acid inhibits induction of c‐Jun protein by ultraviolet radiation that occurs subsequent to activation of mitogen‐activated protein kinase pathways in human skin in vivo	G J Fisher	The Journal of clinical investigation	1998	294	8.91
24	Singlet oxygen mediates the UVA‐induced generation of the photoaging‐associated mitochondrial common deletion	M Berneburg	The Journal of biological chemistry	1999	281	8.52
25	Modulation of skin collagen metabolism in aged and photoaged human skin in vivo	Chung JH	The Journal of investigative dermatology	2001	279	8.45
26	Endogenous UVA‐photosensitizers: mediators of skin photodamage and novel targets for skin photoprotection	Georg T Wondrak	Photochemical & photobiological sciences	2006	267	8.09
27	Photoaging is associated with protein oxidation in human skin in vivo	Sander CS	The Journal of investigative dermatology	2002	260	7.88
28	Solar ultraviolet irradiation reduces collagen in photoaged human skin by blocking transforming growth factor‐beta type II receptor/Smad signaling	Quan T	The American journal of pathology	2004	255	7.73
29	Retinoids in the treatment of skin aging: an overview of clinical efficacy and safety	Siddharth Mukherjee	Clinical interventions in aging	2006	242	7.33
30	Irradiation of Skin with Visible Light Induces Reactive Oxygen Species and Matrix‐Degrading Enzymes	Liebel F	The Journal of investigative dermatology	2012	237	7.18
31	Matrix metalloproteinase‐1 is the major collagenolytic enzyme responsible for collagen damage in UV‐irradiated human skin	Meghan Brennan	Photochemistry and photobiology	2003	235	7.12
32	Antioxidant Properties of Ferulic Acid and Its Possible Application	Zduńska K	Skin pharmacology and physiology	2018	225	6.82
33	Photoaging of human skin	Berneburg M	Photodermatology, photoimmunology & photomedicine	2000	218	6.61
34	UVA‐induced autocrine stimulation of fibroblast‐derived collagenase/MMP‐1 by interrelated loops of interleukin‐1 and interleukin‐6	M Wlaschek	Photochemistry and photobiology	1994	217	6.58
35	Fractional photothermolysis: Current and future applications	Geronemus RG	Lasers in surgery and medicine	2006	216	6.55
36	Natural and Sun‐Induced Aging of Human Skin	Rittié L	Cold Spring Harbor perspectives in medicine	2015	214	6.48
37	Possible involvement of gelatinases in basement membrane damage and wrinkle formation in chronically ultraviolet B‐exposed hairless mouse	Shinji Inomata	The Journal of investigative dermatology	2003	210	6.36
38	Oxidative inhibition of receptor‐type protein‐tyrosine phosphatase kappa by ultraviolet irradiation activates epidermal growth factor receptor in human keratinocytes	Xu Y	Journal of cellular biochemistry	2006	210	6.36
39	Polyphenolic antioxidant (−)‐epigallocatechin‐3‐gallate from green tea reduces UVB‐induced inflammatory responses and infiltration of leukocytes in human skin	S K Katiyar	Photochemistry and photobiology	1999	208	6.30
40	The role of antioxidants in photoprotection: A critical review	Chen L	Journal of the American Academy of Dermatology	2012	207	6.27
41	UV photoprotection by combination topical antioxidants vitamin C and vitamin E	Lin JY	Journal of the American Academy of Dermatology	2003	198	6.00
42	Introduction to skin aging	Tobin DJ	Journal of tissue viability	2017	197	5.97
43	Evaluating cutaneous photoaging by use of multiphoton fluorescence and second‐harmonic generation microscopy	Lin SJ	Optics letters	2005	194	5.88
44	Repeated exposure of human skin fibroblasts to UVB at subcytotoxic level triggers premature senescence through the TGF‐beta 1 signaling pathway	Florence Debacq‐Chainiaux	Journal of cell science	2005	194	5.88
45	Botanical antioxidants in the prevention of photocarcinogenesis and photoaging	Afaq F	Experimental dermatology	2006	190	5.76
46	Ultraviolet A radiation‐induced biological effects in human skin: relevance for photoaging and photodermatosis	J Krutmann	Journal of dermatological science	2000	187	5.67
47	A psychosocial model of sun protection and sunbathing in young women: the impact of health beliefs, attitudes, norms, and self‐efficacy for sun protection	K M Jackson	Health psychology	2000	182	5.52
48	Ferulic acid stabilizes a solution of vitamins C and E and doubles its photoprotection of skin	Fu‐Hsiung Lin	The Journal of investigative dermatology	2005	181	5.48
49	Mechanisms of Photoaging and Cutaneous Photocarcinogenesis, and Photoprotective Strategies with Phytochemicals	Ricardo Bosch	Antioxidants	2015	181	5.48
50	Effects of alpha‐hydroxy acids on photoaged skin: A pilot clinical, histologic, and ultrastructural study	C M Ditre	Journal of the American Academy of Dermatology	1996	178	5.39
51	Green tea polyphenols prevent ultraviolet light‐induced oxidative damage and matrix metalloproteinases expression in mouse skin	Praveen K Vayalil	The Journal of investigative dermatology	2004	174	5.27
52	Photoaging in Asians	Chung JH	Photodermatology, photoimmunology & photomedicine	2003	173	5.24
53	Topical N‐acetyl cysteine and genistein prevent ultraviolet‐light‐induced signaling that leads to photoaging in human skin in vivo	Sewon Kang	Sewon Kang, Jin Ho Chung, Joo Heung Lee, Gary J Fisher, Yian Sheng Wan, Elizabeth A Duell, John J Voorhees	2003	171	5.18
54	The latest on skin photoprotection	González S	Clinics in dermatology	2008	170	5.15
55	Ultraviolet A irradiation stimulates collagenase production in cultured human fibroblasts	Petersen MJ	The Journal of investigative dermatology	1992	169	5.12
56	Prevention of UVB‐induced immunosuppression in mice by the green tea polyphenol (‐)‐epigallocatechin‐3‐gallate may be associated with alterations in IL‐10 and IL‐12 production	S K Katiyar	The Journal of investigative dermatology	1999	167	5.06
57	Singlet oxygen may mediate the ultraviolet A‐induced synthesis of interstitial collagenase	M Wlaschek	The Journal of investigative dermatology	1995	166	5.03
58	Hydrogen peroxide (H_2_O_2_) increases the steady‐state mRNA levels of collagenase/MMP‐1 in human dermal fibroblasts	P Brenneisen	Free radical biology & medicine	1997	163	4.94
59	Biomarkers of Cellular Senescence and Skin Aging	Wang AS	Frontiers in genetics	2018	163	4.94
60	Coenzyme Q (10), a cutaneous antioxidant and energizer	Hoppe U	BioFactors	1999	160	4.85
61	Cutaneous photodamage in Koreans—Influence of sex, sun exposure, smoking, and skin color	J H Chung	Archives of dermatology	2001	159	4.82
62	Enhanced elastin and fibrillin gene expression in chronically photodamaged skin	Bernstein EF	The Journal of investigative dermatology	1994	157	4.76
63	Cutaneous effects of infrared radiation: from clinical observations to molecular response mechanisms	Stefan M Schieke	Photodermatology, photoimmunology & photomedicine	1994	155	4.70
64	Protective effect against sunburn of combined systemic ascorbic acid (vitamin C) and d‐alpha‐tocopherol (vitamin E)	Eberlein‐König B	Journal of the American Academy of Dermatology	1998	150	4.55
65	Aging‐ and photoaging‐dependent changes of enzymic and nonenzymic antioxidants in the epidermis and dermis of human skin in vivo	G Rhie	The Journal of investigative dermatology	2001	149	4.52
66	Percutaneous collagen induction therapy: An alternative treatment for scars, wrinkles, and skin laxity	Matthias C Aust	Plastic and reconstructive surgery	2008	149	4.52
67	Ultraviolet Radiation‐Induced Skin Aging: The Role of DNA Damage and Oxidative Stress in Epidermal Stem Cell Damage Mediated Skin Aging	Uraiwan Panich	Stem cells international	2016	149	4.52
68	Skin photosensitizing agents and the role of reactive oxygen species in photoaging	Dalle Carbonare M	Journal of photochemistry and photobiology. B, Biology	1992	148	4.48
69	Water and protein structure in photoaged and chronically aged skin	Gniadecka M	The Journal of investigative dermatology	1998	147	4.45
70	Endogenous skin fluorescence includes bands that may serve as quantitative markers of aging and photoaging	N Kollias	The Journal of investigative dermatology	1998	147	4.45
71	Topical 5‐aminolevulinic acid combined with intense pulsed light in the treatment of photoaging	Jeffrey S Dover	Archives of dermatology	2005	147	4.45
72	Chronic phototoxicity and aggressive squamous cell carcinoma of the skin in children and adults during treatment with voriconazole	Edward W Cowen	Journal of the American Academy of Dermatology	2010	147	4.45
73	Nano‐lipoidal carriers of tretinoin with enhanced percutaneous absorption, photostability, biocompatibility and anti‐psoriatic activity	Kaisar Raza	International journal of pharmaceutics	2013	147	4.45
74	Chemiluminescent detection and imaging of reactive oxygen species in live mouse skin exposed to UVA	Yasui H	Biochemical and biophysical research communications	2000	145	4.39
75	Isoflavone genistein: Photoprotection and clinical implications in dermatology	Huachen Wei	The Journal of nutrition	2003	140	4.24
76	Skin aging and natural photoprotection	Wulf HC	Micron	2004	139	4.21
77	Effect of topically applied tocopherol on ultraviolet radiation‐mediated free radical damage in skin	B A Jurkiewicz	The Journal of investigative dermatology	1995	138	4.18
78	Potentials and limitations of the natural antioxidants RRR‐alpha‐tocopherol, L‐ascorbic acid and beta‐carotene in cutaneous photoprotection	J Fuchs	Free radical biology & medicine	1998	138	4.18
79	Review of Fractional Photothermolysis: Treatment Indications and Efficacy	Tierney EP	Dermatologic surgery	2009	138	4.18
80	Photoprotection: a Review of the Current and Future Technologies	Wang SQ	Dermatologic therapy	2010	138	4.18
81	Ultraviolet radiation and the skin: Photobiology and sunscreen photoprotection	Young AR	Journal of the American Academy of Dermatology	2017	135	4.09
82	6‐shogaol, a active constiuents of ginger prevents UVB radiation mediated inflammation and oxidative stress through modulating NrF2 signaling in human epidermal keratinocytes (HaCaT cells)	Feng Chen	Journal of photochemistry and photobiology	2019	135	4.09
83	Pomegranate fruit extract modulates UV‐B‐mediated phosphorylation of mitogen‐activated protein kinases and activation of nuclear factor kappa B in normal human epidermal keratinocytes	Farrukh Afaq	Photochemistry and photobiology	2005	132	4.00
84	A Review of the Role of Green Tea ( *Camellia sinensis* ) in Antiphotoaging, Stress Resistance, Neuroprotection, and Autophagy	Mani Iyer Prasanth	Nutrients	2019	132	4.00
85	New insights in photoaging, UVA induced damage and skin types	Claire Battie	Experimental dermatology	2014	131	3.97
86	Review of environmental effects of oxybenzone and other sunscreen active ingredients	Schneider SL	Journal of the American Academy of Dermatology	2019	131	3.97
87	UV‐induced DNA damage initiates release of MMP‐1 in human skin	Kelly K Dong	Experimental dermatology	2008	130	3.94
88	Ethnic skin disorders overview	Halder RM	Journal of the American Academy of Dermatology	2003	129	3.91
89	Childhood exposure to ultraviolet radiation and harmful skin effects: Epidemiological evidence	Green AC	Progress in biophysics and molecular biology	2011	128	3.88
90	Natural products as photoprotection	Saewan N	Journal of cosmetic dermatology	2015	127	3.85
91	Effects of appearance‐based interventions on sun protection intentions and self‐reported behaviors	Heike I M Mahler	Health psychology	2003	127	3.85
92	Fifty years of skin aging	Yaar M	The journal of investigative dermatology. Symposium proceedings	2002	126	3.82
93	Structure activity relationship of antioxidative property of flavonoids and inhibitory effect on matrix metalloproteinase activity in UVA‐irradiated human dermal fibroblast	Gwan‐Sub Sim	Archives of pharmacal research	2007	124	8.27
94	Benefit and risk of organic ultraviolet filters	Nohynek GJ	Regulatory toxicology and pharmacology	2001	121	5.90
95	Fibrillin‐rich microfibrils are reduced in photoaged skin. Distribution at the dermal‐epidermal junction	R E Watson	The Journal of investigative dermatology	1999	121	5.39
96	The effects of aging on the cutaneous microvasculature	Kelly RI	Journal of the American Academy of Dermatology	1995	120	4.59
97	The role of vitamin E in normal and damaged skin	Nachbar F	Journal of molecular medicine	1995	118	4.37
98	A topical antioxidant solution containing vitamins C and E stabilized by ferulic acid provides protection for human skin against damage caused by ultraviolet irradiation	John C Murray	Journal of the American Academy of Dermatology	2008	117	8.56
99	Effects of Collagen and Collagen Hydrolysate from Jellyfish ( *Rhopilema esculentum* ) on Mice Skin Photoaging Induced by UV Irradiation	Yongliang Zhuang	Journal of food science	2009	116	9.54
100	Anti‐photoaging and Photoprotective Compounds Derived from Marine Organisms	Ramjee Pallela	Marine drugs	2010	115	10.33

### 3.1 Year of Publication

**FIGURE 1:**
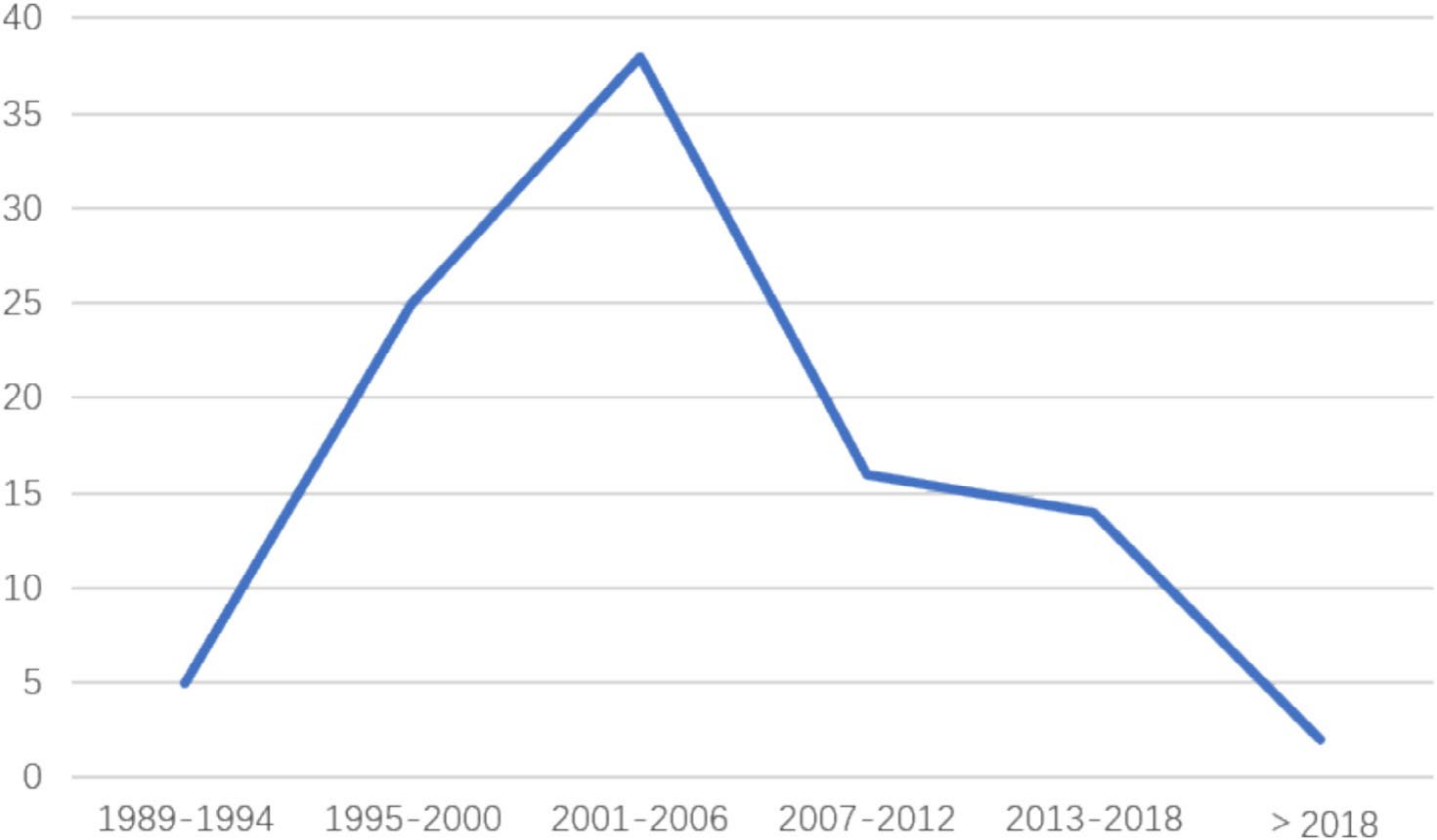
Publication trends of the top 100 most‐cited articles on skin photoaging.

The 100 articles were published between 1989 and 2019. The most influential articles were published from 1995 until 2010 and approximately 33% of articles were published between 2001 and 2005, highlighting the most productive period of influential publications in skin photoaging (Figure 1). The oldest of the included publications, ‘Skin ageing and photoaging—an overview’ by Gilchrest BA, was published in 1989. The most recent article ‘6‐shogaol, an active constiuents of ginger prevents UVB radiation mediated inflammation and oxidative stress through modulating NrF2 signalling in human epidermal keratinocytes (HaCaT cells)’ was published by Feng Chen et al. in 2019.

### 3.2 Citations

The top most‐cited articles on skin photoaging were cited 123 to 1136 times, with a total of 26 855 citations and an average of 269 citations per article. The top most‐cited article was published in 2002 by the University of Michigan in the United States and conducted b**y** Fisher, GJ et al. in the *Archives of Dermatology* entitled ‘Mechanisms of photoaging and chronological skin ageing’. The article received a total of 1136 CC and a CY of 51.63.

### 3.3 Journal of Publications

A total of 56 journals published 100 articles. The *Journal of Investigative Dermatology* and the *Journal of the American Academy of Dermatology* were the two most productive journals, which published 17 and 13 articles, respectively. They both are recognized as high‐quality journals in the field of dermatology with updated impact factors of 6.5 and 13.8, respectively. The journals that contributed three or more publications are presented in Table 2. There was no correlation between the journals' impact factor and their total number of citations in the list. (r = 0.555, p‐value = 0.196). There was also no significant correlation between the journal's impact factor and the number of its articles in the list (r = 0.597, p‐value = 0.157).

**TABLE 2:** Journals that contributed 3 or more publications, with their impact factors and total number of articles in the list.

S.No. Journal Name	Impact Factor	No of articles	Total no. of citations
The journal of investigative dermatology	6.5	17	3838
2 Journal of the American Academy of Dermatology	13.8	13	2813
3 Archives of dermatology	4.789	4	1892
4 Photochemistry and photobiology	3.3	4	845
5 Experimental dermatology	3.6	4	783
6 Free radical biology & medicine	7.4	3	651
7 Photodermatology, photoimmunology & photomedicine	2.6	3	598

### 3.4 Countries, Authors, Institutes and Departments

**FIGURE 2:**
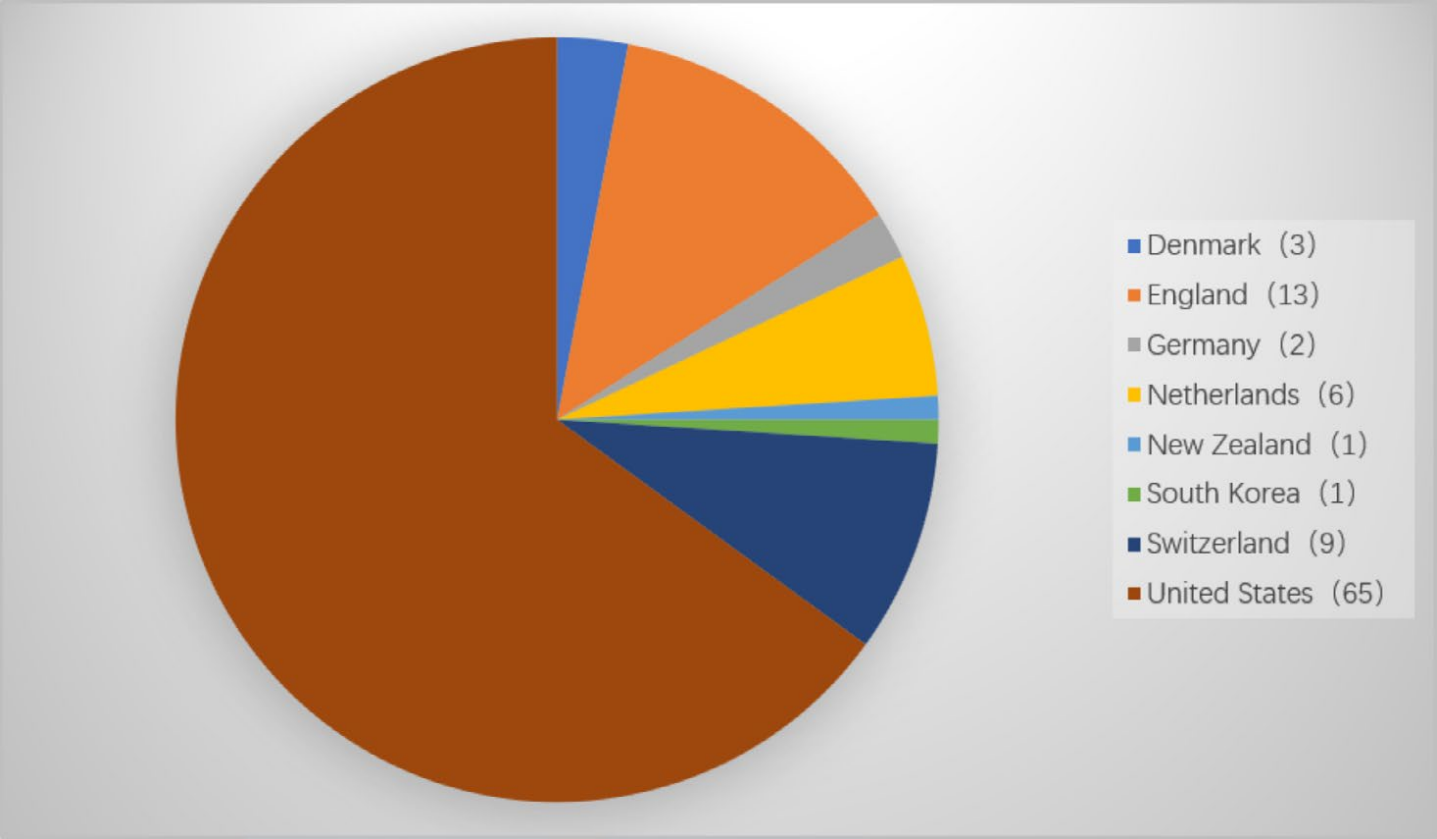
Countries that contributed to the top 100 most‐cited articles.

Overall, the top most‐cited articles originated from nine countries. The United States contributed the most, with a total of 65 articles, followed by England with 13 articles (Figure 2). A total of six authors contributed to more than two articles as the first author. Among them, Fisher GJ was the most productive first author from the Department of Dermatology, University of Michigan Medical School in the United States, publishing a total of three first‐author articles. The other authors include Chung JH (*n* = 2, Department of Dermatology, Seoul National University Hospital), M Wlaschek (*n* = 2, Institute for Physiological Chemistry I, Heinrich‐Heine‐University Dusseldorf), Quan T (*n* = 2, Department of Dermatology, University of Michigan Medical School), S K Katiyar (*n* = 2, Department of Dermatology, Case Western Reserve University, and University Hospitals of Cleveland) and Scharffetter‐Kochanek K (*n* = 2, Department of Dermatology, University of Cologne).

### 3.5 Keywords Analysis and Research Interest

#### 3.5.1 Keywords

**FIGURE 3:**
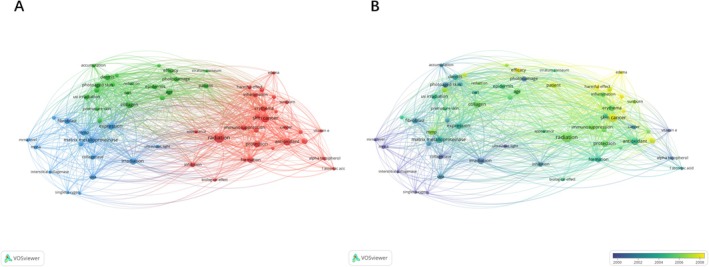
Keywords analysis of the top 100 most‐cited articles. (A) Network visualization map showing cluster analysis of keywords associated with skin photoaging; (B) Network visualization map showing the evolution of keyword frequency over time.

From the top 100 articles on skin photoaging were analyzed using a co‐occurrence network analysis tool of the VOS viewer. We set the minimum count of occurrences at 5. A total of 62 keywords were included and grouped into three clusters: ‘matrix metalloproteinase’, ‘collagen’ and ‘radiation’. In the matrix metalloproteinase cluster, the most prevailing keywords were ‘matrix metalloproteinase’, ‘expression’, ‘irradiation’, ‘fibroblast’ and ‘MMP’. In the collagen cluster, the most prevailing keywords were ‘collagen’, ‘photoaged skin’, ‘dermis’, ‘UV irradiation’ and ‘epidermis’. In the radiation cluster, the most prevailing keywords were ‘radiation’, ‘skin cancer’, ‘antioxidant’, ‘protection’ and ‘oedema’ (Figure 3A).

To determine changes in the research focus over time, we assessed the evolution of the most frequently occurring keywords (Figure 3B). Colors were assigned based on the average number of years the keywords appear in articles. For example, purple keywords appeared more than yellow keywords. The results indicated that research on pathogenesis was a large area of focus during the early years, with a major focus on the prevention and treatment of skin photoaging. For example, recently, there has been a shift towards keywords like ‘efficacy’ ‘photoprotection’ ‘harmful effect’ ‘protection’ and among others.

### 3.6 Article Type and Research Focus

**FIGURE 4:**
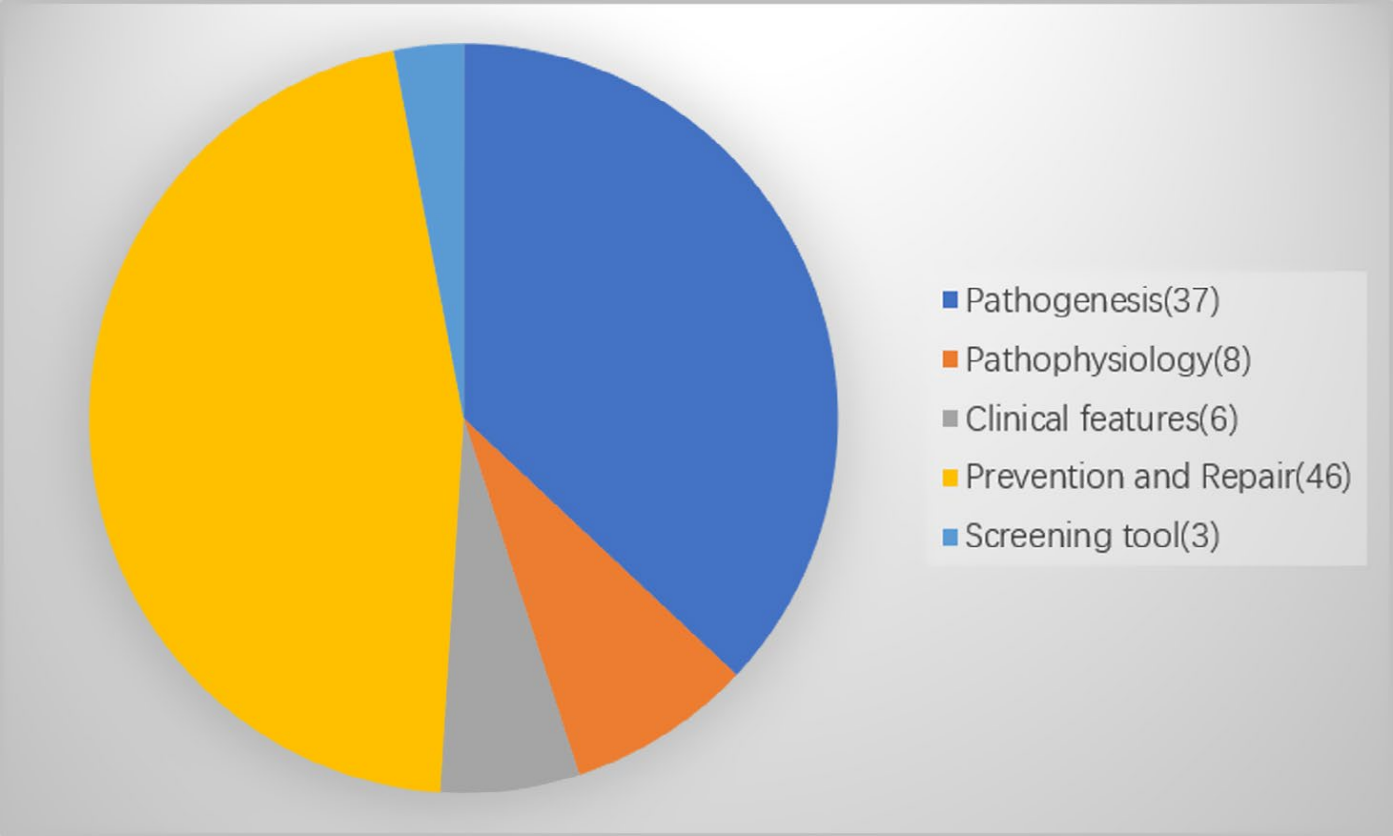
Articles focus of the top 100 most‐cited articles.

Analysis of the top most‐cited 100 articles' titles and abstracts highlighted studies on the following research focuses: pathogenesis (37%), pathophysiology (8%), clinical features (6%), prevention and treatment (46%) and screening tools (3%) (Figure 4). A total of 53 original articles and 47 reviews were included in this study. The pathogenesis of skin photoaging highlighted in the top articles mainly included cellular DNA, RNA, and protein damage; abnormal photooxidative stress pathway; induction of an inflammatory cascade; and extracellular matrix remodeling. The photoprotective therapies against skin photoaging included fractional photothermolysis, adipose‐derived stem cells, topical acids, antioxidants like vitamin C and Vitamin E, and natural products.

## 4 Discussion

This bibliometric analysis of the top 100 most‐cited publications on skin photoaging revealed that the most productive period of influential publications was between 2001 and 2006. Unlike other cutaneous disorders, the research peak occurred later, which could be attributed to the rise in economic empowerment and the public's pursuit of aesthetic appearances. Additionally, the publication of the most cited article in 2002, titled ‘*Mechanisms of photoaging and chronological skin ageing*’, with a total of 1136 citations, published by Fisher GJ et al. [9] also contributed to this increasing trend. The article reviewed the latest information available at that time regarding the molecular pathways that mediate skin photoaging, which became a landmark in skin photoaging research. Since then, there have been numerous high‐quality articles. The second most cited article, entitled ‘*Fractional photothermolysis: A new concept for cutaneous remodeling using microscopic patterns of thermal injury*’ by Manstein D et al. was published in ‘*Lasers in surgery and medicine*’ with a total of 1060 citations [10]. In this article, Fisher GJ et al. demonstrated that repeated and multiple UVR exposure led to sustained elevation of MMPs that degrade skin collagen, which induced skin photoaging. Additionally, treatment with topical tretinoin was reported to reverse the condition.

Our analysis indicated that the majority of the most‐cited articles were published in the *Journal of Investigative Dermatology*, followed by the *Journal of the American Academy of Dermatology*. Their 2022 impact factors are 6.5 and 13.8, respectively, ranking high among various dermatology category journals. This high CC could be attributed to the high equality of articles in these journals or an inherent bias of researchers to select journals with high impact factors for citation. A total of 56 journals published the 100 top most‐cited articles. Apart from dermatology journals, journals focusing on toxicology, pharmacology, cell biology, biochemistry, and molecular biology have also published studies on skin photoaging.

Fisher GJ was the most productive first author from the Department of Dermatology, University of Michigan Medical School in the United States. The total impact factor of his articles reached 2281, and two of the articles ranked first and third, respectively, in the CC. These findings indicate the major contribution of Fisher GJ to the specific field of skin photoaging. The majority of articles originated from the United States, which is consistent with the bibliometric analysis in other fields of dermatology, like rosacea [11], melanoma [12] and Merkel cell carcinoma [13]. Notably, economic growth could be a major reason for this increased contribution.

The results of our co‐occurrence network analysis also indicate a series of keywords closely associated with the pathogenesis, pathophysiology and prevention and treatment of skin photoaging. For example, long‐term UV exposure results in the gradual accumulation of MMPs and a decrease in collagen synthesis by fibroblasts in photoaged skin, ultimately leading to wrinkles. ‘UV’, ‘MMP’, ‘collagen’, ‘fibroblasts’ and ‘photoaged skin’ were identified as the most popular keywords. Keywords related to the photoprotective methods to prevent and treat skin photoaging included ‘antioxidant’ and ‘protection’. Additionally, ‘skin cancer’ is also indicated as one of the most popular keywords as photoaging is closely associated with photocarcinogenesis, including actinic keratosis, basal cell carcinoma and malignant melanoma [14, 15]. The pathogenesis of photoaging and photocarcinogenesis involves direct damage to DNA and indirect damage through inflammation and in vivo immunosuppression, accumulation of MMPs and reduction of collagens [5, 16, 17]. Moreover, studies on pathogenesis were observed to be a large area of focus during the early years, with a large shift towards the prevention and treatment of skin photoaging. Thus, along with the in‐depth investigation of pathogenesis, identifying and developing effective methods to prevent and treat photoaging and photocarcinogenesis is crucial. Furthermore, on analyzing the research focus of the top 100 most‐cited publications, we found that the prevention and treatment of skin photoaging account for a larger number of reported studies, which was consistent with the results of Sun et al. [8], that also indicated a landscape of photoaging, from the bench (pathophysiology), to bedside (treatment, prevention). Among these, physical therapies included fractional photo thermolysis [18, 19], pulsed carbon dioxide laser [20] and intense pulsed light [21], which are clinically applied to reverse skin photoaging. An article reported that percutaneous collagen induction therapy [22], which aims to stimulate collagen production using the normal chemical cascade that ensues after any trauma, has emerged as an alternative treatment for wrinkles and skin laxity. Moreover, various types of acids like retinoic acid [23], ferulic acid, alpha‐hydroxy acids [24] and 5‐aminolevulinic acid [21] have an inhibitory effect on skin photoaging by repairing the epidermal function and stimulating dermal collagen synthesis. A series of articles highlighted the application of oral or topical antioxidants against skin photoaging. Besides the traditional antioxidants like vitamin C, vitamin E [25] and coenzyme Q [10, 26], other natural products including 6‐shogaol, an active constituent of ginger [27], pomegranate fruit extract, polyphenolic antioxidant (−)‐epigallocatechin‐3‐gallate from green tea [28, 29], N‐acetyl cysteine, genistein [30] and isoflavone genistein [31] have been reported to inhibit ultraviolet light‐induced oxidative damage, thereby preventing skin photoaging. Recently, adipose‐derived stem cells have been found to be effective in treating skin photoaging by enhancing the secretion of type I collagen in human dermal fibroblasts [32].

Although we spared no effort in eliminating potential defects in this bibliometric analysis, our study has several limitations. First, bibliometric studies of the top 100 most‐cited articles have their inherent limitations, such as over‐signifying old studies by CC accumulation and under‐signifying recently published impactful articles. It must be noted at some point that this limits its relevance for predicting future research trends. Second, the reliance on CC as a measure of influence can be problematic, as citations do not always reflect the true quality or relevance of a study. CC can be influenced by the publication's journal impact factor, the reputation of the author, or the popularity of the topic at the time of publication. In addition, CC is difficult to quantify public knowledge and familiarity with research findings. Another metrics such as article views or altmetrics, could be applied in future to provide a more holistic evaluation of the studies' impact. Third, the literature used for bibliometric analysis was extracted only from the Web of Science, which might neglect some highly cited articles included in some other databases (such as PUBMED, SCOPUS, and Google Scholar). The analysis of these databases might show different results. Finally, our research was limited as it only included articles published in English. While English‐language studies dominate the field, there may be significant research published in other languages that could provide additional insights into the global understanding of photoaging. Future research could expand the scope to include studies in other languages or at least discuss the potential influence of such publications on the overall landscape. Despite these limitations, this descriptive bibliometric study highlights the most reputable key publications to researchers in the field of photoaging.

## 5 Conclusion

Our study provides a detailed and comprehensive bibliometric analysis of the top 100 most‐cited publications in skin photoaging. Articles published between 1989 and 2019 were included in this study. The majority of the top 100 most‐cited articles were published in the *Journal Of Investigative Dermatology* and the *Journal of the American Academy of Dermatology*. Research on the pathogenesis of skin photoaging was a major focus during the early years, with a gradual shift towards prevention and treatment. The majority of articles focusing on prevention and treatment mainly included physical therapies, various types of topical acids, a series of natural antioxidants, and adipose‐derived stem cells. The 100 included articles originated from nine countries, mostly from the United States. Fisher GJ from the University of Michigan in the United States was the most productive first author in this field, who has published the first and second most‐cited articles. In summary, this study identified the top 100 most‐cited articles in skin photoaging and analyzed their bibliometric characteristics, thereby laying the foundation for further research.

## Author Contributions

Y.T., H.T., Y.F., and J.Y. designed the literature search and wrote the article with input from all authors. Y.T. drafted the manuscript. All authors discussed the results and commented on the manuscript. All authors contributed to the article and approved the submitted version.

## Disclosure

The authors have nothing to report.

## Conflicts of Interest

The authors declare no conflicts of interest.

## Data Availability

The data that support the findings of this study are available on request from the corresponding author. The data are not publicly available due to privacy or ethical restrictions.
